# The Patient Health Questionnaire (PHQ-9) as a tool to screen for depression in people with multiple sclerosis: a cross-sectional validation study

**DOI:** 10.1186/s40359-022-00949-8

**Published:** 2022-11-28

**Authors:** Emily Beswick, Suzanne Quigley, Pamela Macdonald, Sarah Patrick, Shuna Colville, Siddharthan Chandran, Peter Connick

**Affiliations:** 1 Centre for Clinical Brain Sciences, The University of Edinburgh, Edinburgh, Scotland; 2 Anne Rowling Regenerative Neurology Clinic, The University of Edinburgh, Edinburgh, Scotland; 3 The School of Medicine & Veterinary Medicine, The University of Edinburgh, Edinburgh, Scotland; 4 West Scotland Deanery Foundation Programme, NHS Education for Scotland Trainee, Edinburgh, Scotland

**Keywords:** Multiple sclerosis, Depression, Screening, Validity, Reliability, Responsiveness

## Abstract

**Background:**

Depression has a point prevalence of 25% and lifetime prevalence of 50% in people with multiple sclerosis (pwMS). Due to accessibility and brevity, the 9-item Patient Health Questionnaire (PHQ-9) may be a useful tool in clinical practice for screening and monitoring of depressive symptoms in people with MS (pwMS).

**Methods:**

The objective of this study was to evaluate the reliability, validity and acceptability of the PHQ-9 as a screening tool for depressive symptoms in pwMS. PwMS completed online questionnaires at 3 time-points over 4-weeks. The PHQ-9, Multiple Sclerosis Impact Scale (MSIS-29), Centre for Disease Control Health-Related Quality of Life Measure (CDC-HQOL-4) and clinical history.

**Results:**

103 participants completed the PHQ-9 at three time points, 43% were categorised as depressed on at least one response. The PHQ-9 exhibited high internal reliability (Cronbach’s α = 0.89), and test-re-test agreement (ICC 0.89, 95% CI 0.85–0.91). Convergent validity was indicated through positive correlation with the mental health items on the MSIS-29 (r = 0.46 and r = 0.50) and CDC-HQOL-4 (r = 0.79 and r = 0.73) at both assessment points. Positive correlations between the PHQ-9 and the MSIS-29 (r = 0.86 and r = 0.84) and CDC-HQOL-4 (r = 0.55 and r = 0.37) physical symptom sub-scores did not indicate divergent validity. 93% of ratings evaluated the PHQ-9 as “Very” or “Completely” acceptable.

**Conclusion:**

The PHQ-9 is a reliable and valid measure of depressive symptoms in people with MS. Given its accessibility, ease of administration, and acceptability, we recommend the PHQ-9 as a tool to screen for depressive symptoms in people with MS.

**Supplementary Information:**

The online version contains supplementary material available at 10.1186/s40359-022-00949-8.

## Introduction

Multiple sclerosis (MS) affects over 2.3 million individuals worldwide. [1] It is a chronic inflammatory and degenerative disease of the central nervous system characterised by a heterogeneous presentation of sensory, motor, and cognitive impairment. [2] Lifetime prevalence of depression is greater in people with MS (pwMS) than the general population, with point prevalence of 25% [3] and 6.9% [4] respectively. Depression is a major determinant of quality of life in pwMS [5, 6] and is associated with greater levels of fatigue, and reduced adherence to disease-modifying therapy [7]. Suicidal ideation is also present in up to 22% of pwMS, [8] and chronic illness such as MS and depression are significant predictors of future suicide attempts [9, 10]. Despite being both important and treatable, depression is often under-recognised and under-evaluated in clinical practice [11]. Effective screening tools that can be applied widely in clinical practice are therefore urgently required. [12]

Marrie and colleagues evaluated the validity and reliability of six screening measures for depression in pwMS [13]. The Structured Clinical Interview (SCID) for Diagnostic and Statistical Manual Axis I Disorders was used as the reference standard for analyses of criterion validity. Overall results were similar across measures, with the 9-item Patient Health Questionnaire (PHQ-9) demonstrating the highest sensitivity (84%). The wide range of screening tools available mean other factors such as administration time, acceptability to patients, and cost of licensing must also have an impact on tool selection [13].

The PHQ-9 is an attractive candidate to meet clinical requirements. It is a brief, freely available, self-report version of the Primary Care Evaluation of Mental Disorders (PRIME-MD) [14]. The PHQ-9 focuses on evaluation of depressive symptoms from the preceding 2-weeks, with one item screening for suicidal ideation. A systematic issue that may significantly influence the validity of all screening tools is the potential for confounding by symptoms that could reflect either MS or the somatic features of depression. This has been directly addressed for the PHQ-9 by Sjonnessen and colleagues [15] who found that scores were not altered by excluding items on fatigue and concentration.

We also conducted a systematic review [16] of the psychometric properties of the PHQ-9 in pwMS using the National Health Service (NHS) Research and Development Programme framework for the psychometric evaluation and selection of patient reported outcome measures (PROMs). [17] We found that although the appropriateness, convergent validity, and interpretability were established for the PHQ-9 when applied in pwMS, no data was available on the psychometric properties with respect to internal (consistency) or external (test-retest) reliability, acceptability, feasibility, or responsiveness.

## Methods

### Aim and design

Our current study aimed to address the gap in knowledge on the psychometric properties of the PHQ-9, evaluating whether the PHQ-9 is a screening tool for depression in pwMS that can be recommended for use in clinical practice.

The objective of this study was to explore the acceptability of the PHQ-9 as a measure of depressive symptoms to a group of people with multiple sclerosis and to evaluate the psychometric properties of the PHQ-9 in this population.

We report data from a cross-sectional validation study of 103 community dwelling pwMS. All participants completed three study assessments remotely within a 4-week period using online or paper questionnaires (Table 1). Ethical approval was provided by South East Scotland Research Ethics Committee 02 on 20th March 2017.

### Participant characteristics

Participants were recruited through advertisements on the University of Edinburgh Anne Rowling Regenerative Neurology Clinic website and Facebook page, referrals from local clinical team members, and direct invitation to pwMS on a national research registry (Rowling CARE; https://rowling-care.org.uk/). Eligibility criteria were a diagnosis of multiple sclerosis, aged ≥ 16, resident in the UK, and willing and able to provide informed consent. Recruitment occurred between 20/10/2018 and 20/05/2019. All participants provided written informed consent prior to completing the questionnaires.

### Study materials

Participants reported their age, sex, ethnicity, and number of years in formal education. They were also asked to record their disease course (if known), current treatment, year of diagnosis, and year of symptom onset. Participants also responded to questions on their mental health; specifically historical and current diagnoses of depression, treatments for depression, and other mental health conditions.

Each participant completed the Patient Health Questionnaire (PHQ-9), 29-item Multiple Sclerosis Impact Scale (MISIS-29), a 5-point Likert rating of PHQ-9 acceptability, and 4-item Healthy Days Core Module of the Centre for Disease Control and Prevention Health Related Quality of Life (CDC HRQOL-4) [14, 18, 19]. The PHQ-9 includes 9-item requiring responses of 0 (not at all) to 3 (nearly every day) to assess the occurrence of depressive symptoms over the last two weeks. It has 8 items on depressive symptoms and 1 focused on suicidal ideation. Total scores range from 0 to 27, with published thresholds available to classify the burden of depressive symptoms [20]. A threshold score of 10 or higher is considered to indicate ‘mild’ depression, 15 or higher indicates ‘moderate’ depression, and 20 or higher ‘severe’ depression. A threshold score of 15 or more is typically used in clinical settings as a potential diagnostic indicator.

The MSIS-29 contains 20 items focusing on the physical impact of MS on an individual and their ability to complete activities of daily living (ADLs), with 9 items addressing the psychological impact. All items focus on the impact of MS on everyday life over the past two weeks, questions have 5 response levels ranging from 1 (not at all) to 5 (extremely) and higher summed scores indicate greater impact on daily function. The MSIS-29 has high internal consistency (Cronbach’s alpha ≤ 0.91) and high test–re-test reliability (ICC ≤ 0.87) [19].

The CDC HRQOL-4 is a brief self-report measure of participants’ health perception. Respondents are required to rate their general health from 1 (excellent) to 5 (poor) and complete 3 questions on the number of days different aspects of their health were “bad” and impacted upon their daily lives. The Cronbach’s alpha value of the CDC HRQOL-4 has been reported as 0.76 [21], with a value of ≥ 0.7 or 0.8 indicating good reliability [25].

Acceptability of the PHQ-9 to participants was explored using a single question ‘How acceptable did you find the PHQ-9 questionnaire?’ with a five point Likert rating scale offering potential responses ranging from lesser to greater acceptance: Not At All, Slightly, Moderately, Very or Completely, with an option for qualitative feedback.

People with MS who opted to participate received paper questionnaires or an email link to the questionnaire series on SurveyMonkey ®. Participants were required to complete questionnaires at 3 time points, with 2 weeks between each assessment point (Table 1).

**Table 1:** Study Assessment Schedule

	Baseline Assessment	2-Week Assessment	4-Week Assessment
**Informed Consent**	X		
**Participant demographics, disease characteristics and mental health history questionnaire**	X		
**Patient Derived Disease Steps Scale**	X		
**PHQ-9**	X	X	X
**Acceptability rating of the PHQ-9**	X		X
**MSIS-29**	X		X
**CDC-HQOL-4**	X		X

### Rationale for measure selection

To evaluate the criterion validity of the PHQ-9 as a potential assessment for evaluating depressive symptoms in people with MS we compared the PHQ-9 with the MSIS-29 and the CDC-HRQOL. Convergent validity was evaluated using the mental health sub-scale on the MSIS-29 and days affected by mental health in a month on the CDC-HRQOL. Divergent validity was evaluated using the physical health sub-scale on the MSIS-29 and days affected by physical health in a month on the CDC-HRQOL. These measures were selected as they evaluate both mental and physical health within the same assessment, enabling us to keep assessment times to a minimum that is of paramount consideration in MS research as participants often experience fatigue, and we aimed to optimize retention across the three assessment points.

Depression frequently emerges as one of the most important predictors of health-related quality of life, with worse quality of life reported using the CDC-HRQOL by people with MS with clinical diagnoses or self-reported symptoms of depression [22]. Physical symptoms and disability status are also strong predictors of health-related quality of life, both factors affected by depression and mental health [6]. The MSIS-29 considers the presentation of psychiatric symptoms within the context of the physical disability often experienced by those with MS, particularly useful for considering convergent validity of a non-disease specific depression measure.

Using scales such as the MSIS-29 and CDC-HRQOL that contain both physical and mental health sub-scales enables us to explore the interaction between depression, physical symptoms of MS and health-related quality of life in our sample.

### Management of clinically significant scores

Once informed consent had been provided, the participant’s primary care physician was informed of their participation. All participants were also required to provide consent for contact with their primary care physician if clinically significant scores were identified during the study. Clinical significance was defined as a PHQ-9 total score of ≥ 15 or a score of ≥ 1 on the PHQ’s suicidality item.

### Analysis plan

Planned statistical analysis was defined by the Consensus-based Standards for the selection of health Measurement Instruments (COSMIN) proposed guidelines [23]. Participant characteristics were summarised using frequency for categorical variables and mean (standard deviation [SD]) or median (interquartile range [IQR]) for continuous variables. External (test retest) reliability was evaluated by intraclass correlation coefficient (ICC) of repeated administration scores. To evaluate reliability we used establish cut-off values for the ICC, < 0.5 indicating poor reliability, 0.5–0.75 moderate, 0.75–0.90 good and > 0.90 indicate excellent reliability [24]. Internal reliability (consistency) was evaluated by Cronbach’s α with criteria based on a value of ≥ 0.7 or 0.8 indicating good reliability [25]. Convergent validity of the PHQ-9 was evaluated by correlational analyses with the psychological sub-score of the MSIS-29, and CDC-HQOL-4 data on days affected by mental health. Divergent validity was evaluated by correlational analyses with the physical sub score of the MSIS-29, and CDC-HQOL-4 data on days affected by physical health. Both correlational analyses for convergent and divergent validity were evaluated using established criterion for effect size: 0.10–0.30 is considered a smaller effect, whereas 0.30 to 0.50 a larger effect sizes for a correlation [25]. Acceptability was tested against a criterion of > 95% of participants rating the PHQ-9 as ‘very acceptable’ or ‘completely acceptable’. Responsiveness was analysed using exploratory correlational analyses of the change scores across participants and time-points.

### Sample size calculation

The required number of participants was based upon requirements to provide sufficient power to assess the study objectives. Eigenvalues of approximately 3.5 to 4.42 have been reported for the PHQ-9’s first principal component [26]. To provide an unbiased estimate of Cronbach’s alpha, a sample size of 100 participants was therefore necessary [27]. For external reliability analysis, assuming a two tailed α = 0.05 with three observations per participant (n = 3), the ICC could be estimated with a confidence interval width (ω = 0.2) at the following levels (p = 0.9, 0.8, 0.7 and 0.6) with a sample size (к) of 11, 36, 67 and 100 respectively [28].

## Results

**Fig. 1:**
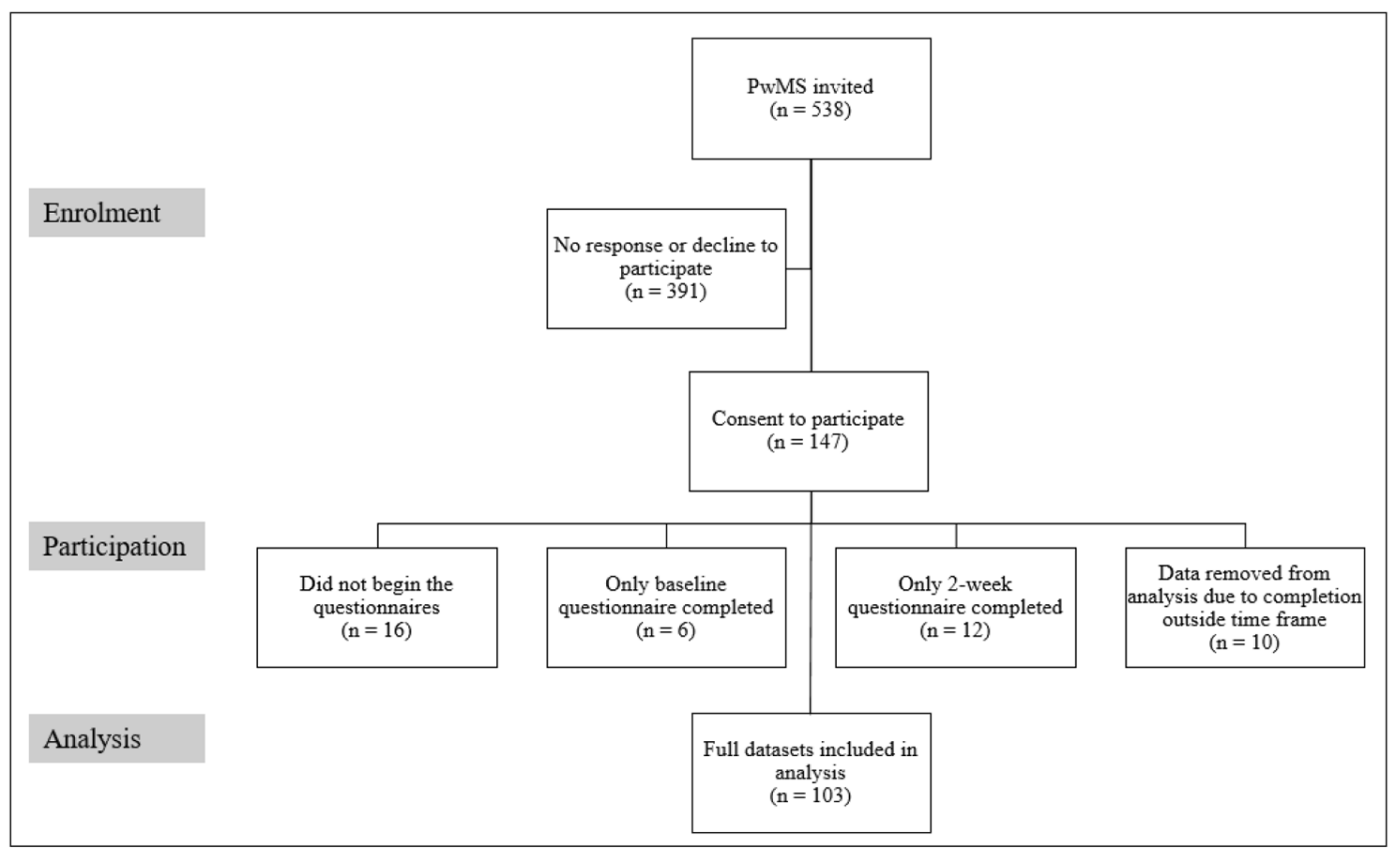
Participant Inclusion

We contacted 538 people with MS, 147 (27%) of whom consented to participate (Fig. 1). 16 of the 147 consented participants did not begin the study. Data from 18 participants was removed, as they did not complete all three study assessment visits. Data from 10 participants was excluded from analysis as responses were provided outside the 2-week period between each stage of data collection (responses < 7 or > 21 days apart). Analysis was undertaken on the 103 complete datasets using R Studio Version 1.2.5019.

### Participants

The cohort was 78% female (Table 2), reflecting typical sex-specific prevalence of MS in the wider population (F:M = 3:1) [2]. However MS disease course was biased towards relapsing-remitting MS, with 66% of participants having relapsing-remitting, 23% secondary-progressive, and 8% primary progressive [2]. 35% of the sample had received a diagnosis of depression at some point in their life, 50% of whom were currently undergoing treatment for depression. Of the 309 responses to the PHQ-9 that were completed by 103 participants at 3 time-points, 42% of total (depression) scores were categorised as ‘Normal’, 34% ‘Mild’, 15% ‘Moderate’, 6% ‘Moderate to Severe’, and 3% ‘Severe’ (Table 3).

**Table 2:** Demographics of Participants

Characteristic	Value
Sex, n (%)	
*Female*	80 (78)
*Male*	23 (23)
Age at enrolment, mean (SD)	50 (11.64)
Race, n (%)	
*White*	102 (99)
*Mixed*	1 (1)
Number of years in full-time education, mean (SD)	18 (3.81)
Sub-type of diagnosis, n (%)	
*Relapsing Remitting Multiple Sclerosis (RRMS)*	68 (66)
*Secondary Progressive Multiple Sclerosis (SPMS)*	24 (23)
*Primary Progressive Multiple Sclerosis (PPMS)*	8 (8)
*No data*	3 (3)
Number of years between symptom onset and diagnosis, mean (SD)	4 (5.91)
Previous diagnosis of depression, n (%)	36 (35)
Currently on treatment for depression, n (%)	18 (17)
Treatments for current depression (n = 18), n (%)	
*Medication*	18 (100)
*Lifestyle modification*	5 (28)
*Psychological intervention*	4 (22)
*Alternative or complimentary therapies*	1 (6)
Diagnosis of other mental health condition (n = 8), n (%)	
*Anxiety*	5 (63)
*Anorexia nervosa*	1 (12.5)
*Anxiety and PTSD*	1 (12.5)
*Complex PTSD*	1 (12.5)

**Table 3:** Number of Respondents in Each Scoring Category for Three Time-Points of PHQ-9 Completion

PHQ-9 Total Score	Interpretation of Total Score	Time Point of Completion		
		*Baseline*	*2-Week*	*4-Week*
0–4	*Normal*	44 (43)	46 (45)	40 (39)
5–9	*Mild*	33 (32)	33 (32)	40 (39)
10–14	*Moderate*	14 (14)	17 (17)	14 (14)
15–19	*Moderate to severe*	7 (7)	4 (4)	8 (8)
20–27	*Severe*	5 (5)	3 (3)	1 (1)

* () Represents percentage of the 103 total respondents

### Reliability

The 9-Item Patient Health Questionnaire (PHQ-9) showed high internal reliability (consistency) for item endorsement (α = 0.89), based on the criteria of a Cronbach’s alpha value of ≥ 0.7 or 0.8 indicating good reliability [29]. External reliability, assessed using intraclass correlation coefficient ICC2k (two-way random effects model, type = mean of k raters and definition of the important relationship = consistency), also indicated a high level of stability between respondents’ scores over the three assessment points. Based on current interpretation criteria, [24] the test-retest reliability of the summed PHQ-9 depression scores showed good agreement across the three time points with an ICC2k of 0.89 (95% CI 0.85–0.91), with values between 0.75 and 0.90 indicating good reliability and 0.90 or above indicating excellent reliability [24].

### Validity

Convergent validity was assessed by Pearson correlational analysis with the 9-item psychological sub-score of the Multiple Sclerosis Impact Scale, and the CDC-HRQOL-4 data on the number of days affected by poor mental health. The total scores of the PHQ-9 and the mental health sub-score of the MSIS were moderately positively correlated at the baseline time-point (r = 0.46), and 4-week time-point (r = 0.50). The total scores of the PHQ-9 and the days affected by mental health sub-score of the CDC-HQOL were strongly positively correlated at the Baseline time-point (r = 0.79), and 4-week time-point (r = 0.73). Both convergent and divergent validity were evaluated using established criterion for effect size: 0.10–0.30 is considered a smaller effect, whereas 0.30 to 0.50 a larger effect sizes for a correlation [25].

Divergent validity was assessed by Pearson correlational analysis of the PHQ-9 scores with the physical sub-score of the Multiple Sclerosis Impact Scale, and the CDC-HRQOL-4 data on the number of days affected by poor physical health. The total scores of the PHQ-9 and the physical health sub-score of the MSIS were strongly positively correlated at the baseline time-point (r = 0.86), and 4-week time-point (r = 0.84). The total scores of the PHQ-9 and the days affected by physical health sub-score of the CDC-HQOL were moderately and weakly positively correlated at the Baseline time-point (r = 0.55), and 4-week time-point (r = 0.37), respectively.

### Acceptability

Acceptability ratings were evaluated against the criterion of > 95% of responses rating the scale as “Very Acceptable” or “Completely Acceptable”. This criterion was not met, as 93% of responses met this level. 103 participants rated acceptability of the PHQ-9 twice, resulting in 206 data points. 30% (n = 62) of responses rated the PHQ-9 as “Very Acceptable”, 63% (n = 130) at “Completely Acceptable”, “Moderately Acceptable” 5% (n = 10) and 1.5% (n = 3) for “Slightly Acceptable. One participant rated the PHQ-9 as “Not at All Acceptable”. In addition, we explored the responses from all participants on their first rating of the PHQ-9, when 92% of participants rated it as “Very Acceptable” or “Completely Acceptable”. 92 participants (89%) rated the PHQ-9 as “Very Acceptable” or “Completely Acceptable” at both time-points.

Of 103 respondents who rated the PHQ-9 acceptability twice (baseline and 4-Week assessments), 34% (n = 35) were inconsistent in their rating responses. This is a well-recognised phenomenon with self-report Likert scales when used to provide feedback on a scale, as the categories are subjective, open to variable interpretation, and highly reductive with regards the construct of ‘acceptability’. To therefore supplement this quantitative data we also invited participants to comment on the PHQ-9 with any areas they found unsuitable; a full list of the comments provided can be found in Table 4 (with potentially identifiable information removed). Sixteen participants provided comments expressing negative feedback or suggestions for changes, categorised as limited number of response options (n = 12, 75%), querying overlap with MS symptoms (n = 2, 12.5%), or highlighting mood symptoms previously ignored by respondents (n = 2, 12.5%).

**Table 4:** Participants’ Feedback on Acceptability of the PHQ-9

Participant	Rating 1	Rating 2
***PHQ002***	This is a confusing question. What does ‘acceptable’ mean? I don’t mind answering the questions, but then again I wasn’t ticking ‘nearly every day’ for every statement so I might feel it’s more intrusive if that was the case. Q16 is also not very well designed. The scale should have a mid-point and a don’t know option!	
***PHQ004***	Not enough range between ‘1’ and ‘several days’. Also, more choice between ‘thoughts of being dead or hurting myself’ which might include feeling a range of guilt rather than wanting to die.	
***PHQ010***	Would like another option for 1 or 2 days. Several days feels like too many but 1 is not true.	For some things I needed a category of 1 or 2. 1 isn’t the case but ‘several days’ feels like too much.
***PHQ023***	Question 5 and 6 about depression doesn’t allow for context which may affect the data you’re collecting and your results. E.g. I am on medicine for depression but the depression is a result of fatigue. So my answers to questions D) and further questions may not be correlated to ‘usual/classic’ symptoms of depression. Hopefully this is ok for your survey.	
***PHQ028***		Asking if you are better or dead or hurting yourself is a very grim thought to think about and put yourself in - not very appropriately.
***PHQ032***	there is not a choice for occasionally. I think it made me more depressed than I’ve ever been.	It seems to accentuate the negative and filling it in as best as I could makes my me sound more “down” that I actually am!
***PHQ035***	if you answer no, then you should skip to the next appropriate question	
***PHQ041***	Two points. 1. No opportunity to refer to other possible current “depression " triggers − 2. Question 16 did not offer a choice between frequency of “1 " and “several days”. Personally 3–12 h would have suited frequencies which I necessarily chose as “several days”. Possibly nitpicking, but the difference between 1 and for a short period I should have thought is more significant than a difference of degree.	
***PHQ045***	I think there needs to be another option in the choices of “occasionally” in between 1 and several days - I felt that sometimes I wanted to answer between the 2 options	the survey needed a fifth option, between “1” and “several days” - occasionally. there were some questions that I felt I wanted to say I had experienced the situation over the 2 weeks, but several days seemed too frequent.
***PHQ047***		It was acceptable but as there were only 4 points to the scale I often had to settle for the nearest one when I perhaps would have preferred more options
***PHQ060***	Would have liked a ‘one or two days’ option.	Think I said before, but think there should be an option between ‘1’ and ‘Several days’; e.g. ‘One or two days’.
***PHQ063***	Does not pick up variability in symptoms (e.g. fatigue) & resulting effects on mood, ability to cope, sense of isolation.	Not detailed enough to pick up complex interactions/connections between physical state of health & mood/mental health (e.g. effects of isolation due to physical state on mood).
***PHQ073***	May be easily attributed to busy schedule rather than MS/depression -- some way of discriminating between the two might be useful.	
***PHQ076***	there is a huge gap between ‘1’ and ‘several days’. for example, I felt tired for a short time, not even one day though.	I find that I experience the feelings described occasionally, for very short periods of time, so until this 3rd questionnaire I have always answered 1. This is because I do not suffer any of them for whole days, or even parts of days. Rather, only a few minutes at a time. I felt I should register these feelings so have used the first column this time. It would have been more helpful to have possible selections such as ‘ever’, ‘often’, ‘frequently’ etc.
***PHQ087***	Need more options or space to qualify statement	
***PHQ098***	4 years of psychotherapy means no question is odd or bad!!	No questions inappropriate - more that it’s so very general
***PHQ107***	Range of days in D would be helpful. One day is neither ‘several’ or ‘1’	
***PHQ111***	Is there a suggestion that I should feel depressed?	
***PHQ118***	Every day should also be an option An additional question asking how much the way we are feeling is because of our MS would be 2	
***PHQ123***	I feel the categories for the current mood question were too wide.	
***PHQ124***		I wondered about the ‘better off dead’ aspect. I think it is different from thoughts of ending one’s life, is this difference intentional? I also think it is quite suggestive to people with a new diagnosis, or a recent relapse, for example. Perhaps thoughts that life is not worth living would be a better way to phrase this. I just find it slightly insensitive.
***PHQ125***		How certain questions are asked. For example, when asking about my general health does this mean excluding my MS?
***PHQ139***		It made me think that should I have these thoughts.

### Responsiveness

The change in the total scores of the PHQ-9 and the mental health sub-score of the MSIS, and the change of the PHQ-9 with days affected by mental health sub-score of the CDC-HQOL were both moderately positively correlated, both at (r = 0.32).

## Discussion

This study evaluated the psychometric properties and acceptability of the Patient Health Questionnaire-9 (PHQ-9) to screen for depressive symptoms in people with multiple sclerosis (MS). The mean scores for depression on the PHQ-9 were higher than would be expected in the general population, consistent with current literature on the elevated prevalence of depressive disorders in people with MS. Using the established criterion of a PHQ-9 score ≥ 10, yielding a sensitivity of 88% and specificity of 88% for major depression [20], 40% of respondents were categorised as depressed at a minimum of one time-point of PHQ-9 completion.

The PHQ-9 had good internal and external reliability. Convergent validity was evidenced by moderate to strong positive correlations of PHQ-9 scores to the psychological sub-score of the Multiple Sclerosis Impact Scale, and to CDC-HRQOL-4 data on the number of days affected by poor mental health. External reliability indicates that the PHQ-9 was suitable for use across repeated assessment time points, with scores remaining relatively stable across multiple administrations.

Evidence of divergent validity was not observed in our study, with strong positive correlations between the PHQ-9 and the physical sub-score of the Multiple Sclerosis Impact Scale (MSIS-29), and weaker positive correlation between the PHQ-9 and CDC-HRQOL-4 data on the number of days affected by poor physical health. However, mental health for people with chronic conditions is often closely associated with variation in their physical symptoms [30], and we therefore do not interpret these findings as raising significant concerns about construct validity of the PHQ-9. Indeed our findings are supportive of the clinical view that pwMS who have greater physical disability are at higher risk of depression, potentially representing a group for whom screening tools are of greatest value. Recognising the close association between physical and mental health can inform our decision-making regarding clinical interventions to manage depressive symptoms in people with MS. Focusing on alleviating the burden of physical symptoms and effective management strategies, offers another potential therapeutic strategy to support people with MS affected by depression.

The exploration of divergent validity using a measure of physical symptoms is a limitation of this study, as mental and physical symptoms often have significant overlap in chronic conditions. Further research into the suitability of the PHQ-9 may benefit from considering additional aspects of MS that are less likely to vary with mental health to evaluate the tool’s divergent validity. In the current study, we opted to assess divergent validity with associations between mental and physical symptoms to evaluate the overlap between these two aspects of health.

The PHQ-9 did not meet our pre-specified acceptability criterion that > 95% participants endorsed ‘Completely’ or ‘Very acceptable’; 93% of participants endorsed these responses. The most frequent criticism was that the number of response categories was too limited. This is a reasonable criticism of any ordinal instrument, but given the requirement for any screening instrument to be suitable for use at scale, there is a pragmatic trade-off between necessary brevity and loss of detail. We therefore interpret these user comments as a reminder about the requirement for clinical judgement in the interpretation of PHQ-9 scores, and not a fundamental barrier to use in the role of a screening instrument. The second most frequent criticism was a lack of focus on MS symptoms. Most concerning was the rare criticism that assessment of depressive symptomatology may *cause* unnecessary rumination on unpleasant phenomena, or potentially be ‘self-fulfilling’ for emergence of depression. Noting the participant’s comments together with the high level of endorsement for ‘completely’ or ‘very acceptable’ responses, our overall interpretation is that the PHQ-9 exhibits satisfactory acceptability for application in clinical practice.

Our exploratory analysis of responsiveness did not support definitive conclusions about the performance of the PHQ-9. Moderate positive correlations were seen between change in PHQ-9 scores and changes in both the CDC-HQOL days affected by mental health and the MSIS psychological sub-scale. However, the minimal extent of change seen due to the short time course of our study and lack of therapeutic intervention were significant limitations. Future work in this area must focus on the concept of responsiveness, considering how we can establish if the PHQ-9 is able to detect those changes in depressive symptoms over time that would be crucial in establishing it as a suitable measure [31].

Our study captured a representative sample of pwMS with respect to sex but had over-representation of participants with relapsing-remitting disease. The prevalence of depression is known to be higher in people with progressive MS, therefore this is a key population for screening. Our findings should therefore be interpreted with caution when extrapolated to this group and we would welcome further specific assessment in that important MS population. We also assume that PHQ-9 psychometric properties are comparable between questionnaires completed using paper or online methods. Although we do not expect substantial differences between these modes of administration, we cannot exclude that possibility.

Finally, the value of a screening instrument also lies in its predictive utility to identify people who require further assessment. Specifically, we cannot infer that the usual thresholds of impairment for the PHQ − 9 are suitable for such application in the MS population, as they were defined using participants without neurological conditions. Future work should therefore focus on establishing the utility of the PHQ-9 to identify clinically significant depressive symptoms in people with MS and the optimum impairment cutoffs for this objective. In addition, exploring divergent validity of the PHQ-9 to evaluate depressive symptoms independent from physical symptoms, as these may be reasonably expected to correlate with an individual’s mental health.

Item response theory is an additional area of interest for future work exploring the suitability of the PHQ-9 to evaluate depressive symptoms in pwMS. Previous studies have indicated that analyses based on item response theory have been useful to evaluate the PHQ-9 in primary care [32] or for people with diagnoses of an affective disorder [33], and may be further able to inform our decisions regarding the usefulness of PHQ-9 in pwMS.

## Conclusion

The social and personal cost of unrecognized, and therefore unmanaged, depression in people with multiple sclerosis cannot be overstated. Depression has consistently been associated with impaired functioning, increased disability, and significant emotional distress. Effective management of depression can reduce these negative effects, therefore accurate identification of which individuals will benefit from depression management strategies can ultimately improve patient care. Whilst self-report rating scales are not sufficient in isolation for psychiatric diagnosis, their clinical utility to highlight potential cases of depression in this chronically ill population is evident.

The PHQ-9 has shown to be psychometrically robust and acceptable to the intended screening population. With previous research on the comparability of screening measures indicating generally good and comparable psychometric properties utilised in the MS population, the focus shifts to ease and feasibility of use when considering clinical applicability. The PHQ-9 is available in the public domain and at only nine items, is brief to complete and with minimal burden to the respondent. We found the PHQ-9 to be a reliable, valid and acceptable measure and we therefore recommend it for use in a clinical context to screen for depression and suicidal ideation in people with MS.

## Electronic supplementary material

Below is the link to the electronic supplementary material.

Supplementary Material 1

## Data Availability

All data generated or analysed during this study are included in this published article and its supplementary information files.

## List of abbreviations

MS: Multiple Sclerosis
PwMS: People with Multiple Sclerosis
PHQ-9: Patient Health Questionnaire
MSIS-29: Multiple Sclerosis Impact Scale
CDC-HQOL-4: Centre for Disease Control Health-Related Quality of Life Measure
SCID: Structured Clinical Interview
PRIME-MD: Primary Care Evaluation of Mental Disorders
NHS: National Health Service
PROM: Patient-Reported Outcome Measure
ADL: Activities of Daily Living
COSMIN: Consensus-based Standards for the Selection of Health Measurement Instruments
IQR: Inter-quartile range
ICC: Intraclass Correlation Coefficient

## References

[1] Organization WH, *Atlas: multiple sclerosis resources in the world 2008* 2008.

[2] Compston A, Coles A (2008). Multiple Scler Lancet.

[3] Marrie RA (2015). The incidence and prevalence of psychiatric disorders in multiple sclerosis: a systematic review. Multiple Scler J.

[4] Wittchen H-U (2011). The size and burden of mental disorders and other disorders of the brain in Europe 2010. Eur Neuropsychopharmacol.

[5] Fernández-Jiménez E, Arnett PA (2015). Impact of neurological impairment, depression, cognitive function and coping on quality of life of people with multiple sclerosis: A relative importance analysis. Multiple Scler J.

[6] Marrie RA (2012). Cumulative impact of comorbidity on quality of life in MS. Acta Neurol Scand.

[7] Mohr DC (1997). Treatment of depression improves adherence to interferon beta-1b therapy for multiple sclerosis. Arch Neurol.

[8] Feinstein A (2011). Multiple sclerosis and depression. Multiple Scler J.

[9] Nielson B, Wang A, Bille-Brahe U (1990). Attempted suicide in Denmark. Acta Psychiatr Scand.

[10] Eliasen A, Dalhoff KP, Horwitz H (2018). Neurological diseases and risk of suicide attempt: A case–control study. J Neurol.

[11] Mohr DC (2006). Treatment of depression for patients with multiple sclerosis in neurology clinics. Multiple Scler J.

[12] Kanner AM, Barry JJ (2003). The impact of mood disorders in neurological diseases: should neurologists be concerned?. Epilepsy Behav.

[13] Marrie RA (2018). The validity and reliability of screening measures for depression and anxiety disorders in multiple sclerosis. Multiple Scler Relat disorders.

[14] Spitzer RL (1999). Validation and utility of a self-report version of PRIME-MD: the PHQ primary care study. JAMA.

[15] Sjonnesen K (2012). Evaluation of the 9–item patient health questionnaire (PHQ–9) as an assessment instrument for symptoms of depression in patients with multiple sclerosis. Postgrad Med.

[16] Patrick S, Connick P. *Psychometric properties of the PHQ-9 depression scale in people with multiple sclerosis: A systematic review*. PloS one, 2019. 14(2). 10.1371/journal.pone.0197943 PMC6380554 30779803

[17] Fitzpatrick P (1998). Evaluating patient-based outcome measures for use in clinical trials. Health Technol Assessment.

[18] Newschaffer C. Validation of Behavioral Risk Factor Surveillance System (BRFSS) HRQOL measures in a statewide sample. Atlanta: US Department of Health and Human Services. Public Health Service, Centers for Disease Control and Prevention, National Center for Chronic Disease Prevention and Health Promotion; 1998.

[19] Hobart J (2001). The multiple sclerosis impact scale (MSIS-29) a new patient-based outcome measure. Brain.

[20] Kroenke K, Spitzer RL, Williams JB (2001). The PHQ-9: validity of a brief depression severity measure. J Gen Intern Med.

[21] Yin S (2016). Summarizing health-related quality of life (HRQOL): development and testing of a one-factor model. Popul health metrics.

[22] Berrigan LI (2016). Health-related quality of life in multiple sclerosis: direct and indirect effects of comorbidity. Neurology.

[23] Mokkink LB (2010). The COSMIN study reached international consensus on taxonomy, terminology, and definitions of measurement properties for health-related patient-reported outcomes. J Clin Epidemiol.

[24] Koo TK, Li MY (2016). A guideline of selecting and reporting intraclass correlation coefficients for reliability research. J Chiropr Med.

[25] Cohen J. Statistical power analysis for the behavioral sciences. Academic press; 2013.

[26] Huang FY (2006). Using the patient health questionnaire-9 to measure depression among racially and ethnically diverse primary care patients. J Gen Intern Med.

[27] Yurdugül H (2008). Minimum sample size for Cronbach’s coefficient alpha: a Monte-Carlo study. Hacettepe Üniversitesi eğitim fakültesi dergisi.

[28] Shoukri MM, Asyali M, Donner A (2004). Sample size requirements for the design of reliability study: review and new results. Stat Methods Med Res.

[29] Kline P. A Handbook of Psychological Testing. Routledge (London; 1999.

[30] da Silva AM (2011). Depression and anxiety in a Portuguese MS population: associations with physical disability and severity of disease. J Neurol Sci.

[31] Giordano A (2009). Responsiveness of patient reported outcome measures in multiple sclerosis relapses: the REMS study. J Neurol Neurosurg Psychiatry.

[32] Cumbe VFJ (2020). Validity and item response theory properties of the Patient Health Questionnaire-9 for primary care depression screening in Mozambique (PHQ-9-MZ). BMC Psychiatry.

[33] Adler M (2012). An item response theory evaluation of three depression assessment instruments in a clinical sample. BMC Med Res Methodol.

